# Integrated exome and transcriptome analysis prioritizes MAP4K4 de novo frameshift variants in autism spectrum disorder as a novel disease–gene association

**DOI:** 10.1007/s00439-022-02497-y

**Published:** 2022-12-05

**Authors:** M. Cesana, L. Vaccaro, M. J. Larsen, M. Kibæk, L. Micale, S. Riccardo, P. Annunziata, C. Colantuono, L. Di Filippo, D. De Brasi, M. Castori, C. Fagerberg, F. Acquaviva, D. Cacchiarelli

**Affiliations:** 1 Armenise/Harvard Laboratory of Integrative Genomics, Telethon Institute of Genetics and Medicine (TIGEM), Pozzuoli, Italy; 2 Department of Advanced Biomedical Sciences, University of Naples “Federico II”, Naples, Italy; 3 Department of Clinical Research, Faculty of Health Sciences, Clinical Genome Center and Human Genetics, University of Southern Denmark, Odense, Denmark; 4 Department of Clinical Genetics, Odense University Hospital, Odense, Denmark; 5 Department of Pediatrics, Odense University Hospital, Odense, Denmark; 6 Division of Medical Genetics, Fondazione IRCCS-Casa Sollievo della Sofferenza, San Giovanni Rotondo, Foggia, Italy; 7 Next Generation Diagnostic srl, Pozzuoli, 80078 Naples, Italy; 8 Department of Pediatrics, AORN Santobono-Pausilipon, 80122 Naples, Italy; 9 Department of Translational Medicine, University of Naples “Federico II”, Naples, Italy; 10 SSM School for Advanced Studies, University of Naples “Federico II”, Naples, Italy

## Abstract

The application of next-generation sequencing (NGS) to clinical practice is still hampered by the ability to interpret the clinical relevance of novel variants and the difficulty of evaluating their effect in specific tissues. Here, we applied integrated genomic approaches for interrogating blood samples of two unrelated individuals with neurodevelopmental disorders and identified a novel neuro-pathogenic role for the Mitogen-Activated Protein Kinase 4 gene (MAP4K4). In particular, we identified two novel frameshift variants in coding exons expressed in the blood and neuronal isoforms. Both variants were predicted to generate non-sense-mediated decay. By transcriptome analysis, we simultaneously demonstrated the deleterious effect of the identified variants on the splicing activity and stability of MAP4K4 mRNA. Therefore, we propose MAP4K4 as a novel causative gene for non-syndromic and syndromic neurodevelopmental disorders. Altogether, we prove the efficacy of an integrated approach of exome and transcriptome sequencing in the resolution of undiagnosed cases by leveraging the analysis of variants in genes expressed in peripheral blood.

**Supplementary Information:**

The online version contains supplementary material available at 10.1007/s00439-022-02497-y.

## Introduction

Mitogen-activated protein kinase kinase kinase kinases (MAP4Ks) belong to the mammalian *S. cerevisiae* Sterile 20 (Ste20)-like family of serine/threonine kinases which includes the six paralogous proteins MAP4K1 to MAP4K6. Among them, MAP4K4, also known as hepatocyte progenitor kinase-like/germinal center kinase-like kinase (HGK) and Nck-interacting kinase (NIK), is a well-conserved protein containing 1288 amino acids that structurally includes an N-terminal kinase domain, proline-rich motifs, a coiled-coil domain, a C-terminal hydrophobic leucine-rich citron homology domain (CNH) and two putative caspase cleavage sites (Delpire 2009). Alternative splicing of the MAP4K4 transcript results in five isoforms that display differences in inter-domain composition, while kinase and CNH domains are 100% homologous.

The in vivo roles of MAP4K4 are largely unknown due to the early lethality of Map4k4 whole-body knockout mice (Xue et al. 2001). MAP4K4 is implicated in various physiological cell functions closely associated with the tumor necrosis factor-alpha (TNFα)-induced and c-Jun N-terminal kinase (JNK) signaling pathways. MAP4K4 is an upstream activator of MAPK/JNK, interacting with TAK1 (MAP3K7), MKK4 (MAP2K4), or MKK7 (MAP2K7) (Diener et al. 1997; Su et al. 1997; Taira et al. 2004), and potentially mediating the signal from TNFα.

MAP4K4 is also a modulator of the remodeling of the F-actin cytoskeleton by directly controlling polymerization dynamics and signaling mediators associated with cytoskeleton control. The modulation of the cellular cytoskeleton by MAP4K4 is connected to diseases, such as aberrant angiogenesis and tumorigenesis (Gao et al. 2016; Tripolitsioti et al. 2018).

Several genes involved in the MAP4K4-mediated cascade have been associated with various rare and ultra-rare hereditary disorders. In particular, heterozygous variants in MAP3K7, encoding TAK1, and its major interactor TAB2, cause cardio-spondylocarpofacial syndrome (Goff et al. 2016), cardio-faciocutaneousarticular syndrome (Micale et al. 1928) and fronto-metaphyseal dysplasia (Wade et al. 2016). Deleterious variants in MAP3K1 have been identified in autosomal dominant 46, XY gonadal dysgenesis (Pearlman et al. 2010). Heterozygous variants in MAP2K1/MEK1 and MAP2K2/MEK2 cause cardio-facio-cutaneous syndrome type 3 and 4, respectively (Rodriguez-Viciana et al. 2006). Finally, a single nonsense heterozygous change in MAP4K4 has been reported in a fetus with a congenital heart defect and urinary tract malformations (Vora et al. 2017). Besides such a single case, members of the MAPK4 family have never been associated with Mendelian disorders.

By leveraging the integrated use of exome and transcriptome sequencing, we report de novo, frameshift variants in MAP4K4 in two unrelated individuals with global developmental delay and autism. This study, for the first time, identifies MAP4K4 as a rare association of Mendelian neurodevelopmental disorders in humans.

## Materials and methods

### Patients’ enrollment

Individual 1 and his parents were enrolled at the Department of Pediatrics of the Santobono-Pousilipon Children's Hospital in Naples (Italy) during routine assessment for a delay in the development of neuro-motor milestones and suspicion of autism spectrum disorder. Individual 2 and his parents were enrolled at the Department of Pediatrics of the Odense University Hospital in Odense (Denmark) during routine assessment for developmental delay. This study was in accordance with the Helsinki declaration (1984) and subsequent revisions. The methods were performed in accordance with relevant guidelines and regulations. Both families signed written informed consent for the publication of clinical and molecular data, including photos.

### Exome sequencing

Both families underwent diagnostic evaluation within their clinical centers including exome sequencing analysis, which was performed according to each center's standard practice (detailed protocols are available upon request). In summary, gDNA was extracted with paramagnetic beads technology, fragmented, and ligated to generate a genomics library which was then captured with a capture-based probe covering the hg38 human genome. The resulting library was then sequenced with a paired-end 150 cycles strategy to guarantee a minimal per-base coverage of 30X. All approaches are in line with the latest ACMG guidelines for exome sequencing for pediatric patients (Manickam 2022).

The bioinformatics pipeline followed standard practices to generate a list of annotated variants. Variants were finally classified according to the American College of Medical Genetics and Genomics/Association for Molecular Pathology (ACMG/AMP) criteria (Richards et al. 2015). In particular, the raw data were analyzed by Next-Generation Diagnostic srl Whole-Exome Sequencing pipeline (v1.0), which involves a cleaning step by UMI removal (UMI-tools, v.1.1.1) (Smith et al. 2017), quality filtering (FASTQC, v.) (Babraham Bioinformatics, 2022) and trimming (Trimmomatic, v.0.38) (Bolger et al. 2014), alignment to the human reference genome (hg38) (BWA, v.0.7.17-r1188), removal of duplicate reads and variant calling (Sentieon, v.202011.01) (Freed et al. 2017). Variants were finally annotated by the Ensembl Variant Effect Predictor (VEP) tool (McLaren et al. 2016) (v. 104), and final clinical interpretation including ACMG/AMP rules was performed using Varsome Clinical (v.11.4). (Kopanos et al. 2019)

Variants filtering involved the subsequent removal of: (i) variants with an allele frequency < 0.05 for autosomal recessive genes and < 0.01 for autosomal dominant genes; (ii) variants with borderline allele frequency (0.01–0.05) and annotated as benign or likely benign variants without conflicts in ClinVar (https://www.ncbi.nlm.nih.gov/clinvar/); (iii) variants annotated in ClinVar as a risk factor or histocompatibility allele; (iv) heterozygous variants inherited from an unaffected parent for autosomal dominant genes with complete penetrance in the pediatric age; (v) heterozygous variants in autosomal recessive genes without the simultaneous identification of a second rare variant. The result was a list of apparently de-novo variants in genes with any type of transmission, rare homozygous variants in autosomal recessive genes, and possible rare compound heterozygous variants in autosomal recessive genes with at least one variant annotated as pathogenic/likely pathogenic according to the ACMG/AMP rules. Both MAP4K4 candidate variants were confirmed by Sanger sequencing in independent blood drawn from both the pro-bands and their parents. The variant in Individual 1 was confirmed with the following primers: 5′-CCGAACAAATCCAGAGAAGAG-3′ (forward) and 5′-AGCTTGGCTTGCTCCTTTC-3′ (reverse). The variant in Individual 2 was confirmed with the following primers: 5′-CCTGGTCTGCGGCTGAGATACACAGAGCGACAGAGACATTT-3′ (forward) and 5′-GGAGAGGAAGGAGGGGAGGGGTTATCAT-3′ (reverse).

### Transcriptome sequencing

Independent blood samples drawn were re-collected, and individuals’ samples were harvested in either Tempus Blood RNA Tube (Thermo Fisher) or PAXgene Blood RNA Tubes (Qiagen). RNA was extracted using custom RNA extraction core services using paramagnetic beads technology (Fondazione Telethon spin-off—Next Generation Diagnostic srl). Total RNA was quantified using Qubit 2.0 fluorimetric Assay (Thermo Fisher Scientific). Libraries were prepared from 500 to 1000 ng of total RNA using the NEGEDIA Full Length mRNA-seq service (Next Generation Diagnostic srl), which included stranded library preparation, quality assessment and sequencing on a NovaSeq 6000 sequencing system using a paired end, 150 cycle strategy (Illumina Inc.).

FASTQ reads were analyzed with Next Generation Diagnostic srl proprietary full-length mRNA-seq pipeline (v1.0), which involves quality control, alignment to the reference hg38 human genome and counting steps (Dobin et al. 2013; Li and Dewey 2011). IGV was applied to visualize the BAM file's exon coverage, positional variants and sashimi plots based on sequences mapping across exon junctions (split reads) (Robinson 2011).

RNAseq data for different tissues were downloaded from HPA (https://www.proteinatlas.org/) database (Karlsson et al. 2021; Uhlen et al. 2019). Every cell type belonging to the same tissues or system was divided into main groups (brain, blood, immune system, muscles, male tissues, female tissues, and intestine) and the average value for each isoform was calculated.

## Results

### Clinical reports

**Fig. 1:**
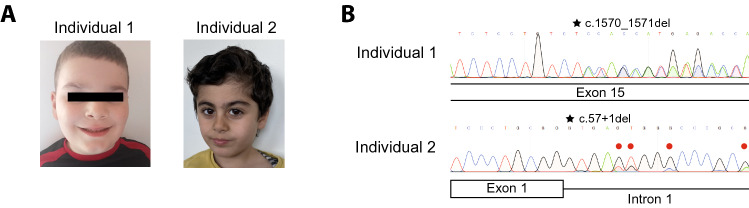
Exome and DNA analyses associate MAP4K4 variants to autism spectrum disorders**. A** Pictures showing the affected individuals. Informed written consent for the images was obtained. **B** Sanger sequencing spectra showing heterozygous variants in exon 15 (Individual 1) and mosaicism of exon 1 (Individual 2). Black stars mark the position of the variants. Red circles in Individual 2 electropherogram indicate representative nucleotides where it is possible to appreciate mosaicism

Individual 1 was a 4-year and 2-month-old boy, the first child of unaffected and unrelated Italian parents. The family history was unremarkable for developmental disorders. He was born at term from an uneventful pregnancy and by elective cesarean section. Birth parameters (38 weeks) were length 49 cm, weight 3070 g, and head circumference 33 cm, all within normal ranges. Apgar score was 6_1_/8_5_/10_10_. Abdominal, kidney, and heart ultrasounds at birth were normal. Psychomotor development was delayed for language and sphincter control. He early underwent a program of speech and psychomotor therapy. Endocrinological workout at 2 years demonstrated hypogonadal hypogonadism (LH < 0.1 mU/mL and FSH 0.5 mU/mL). Physical assessment by the Clinical Geneticist at 4 years and 2 months showed a height of 112 cm (> 99th centile; *Z*-score + 2.126), a weight of 34 kg (> 99th centile; *Z*-score + 5.087), head circumference of 51.5 cm (30th centile; *Z*-score − 0.568), micro-penis and underdeveloped genitalia. Facial features included clinical hypertelorism and sparse eyebrows (Fig. 1A left). There were not any appendicular anomalies and the skin was unremarkable. Neurodevelopmental assessment at 5 years, carried out with the ABI-R, ADOS, and Leiter III scales, concluded for a level 3 autism spectrum disorder with severe communication and social interaction difficulties. FMR1 molecular testing, SNP-array, screening for Angelman/Prader–Willi syndrome by MS-MLPA, targeted NGS for intellectual disability/autism spectrum disorder and hypogonadal hypogonadism, metabolic profile gave negative or normal results. He never performed a brain MRI.

Individual 2 was a 3-year and 8-month-old boy, the first child of unaffected and unrelated Arabic–Kurdish parents. The family history was unremarkable for developmental disorders. He was born at term from an uneventful pregnancy and by natural delivery. Birth parameters (39 weeks) were length 50 cm, weight 3150 g, and head circumference 33 cm, all within normal ranges; Apgar score was 10_1_/10_5_. Psychomotor development was delayed for language, while motor skills were obtained regularly. Physical assessment at 3 years and 8 months showed a height of 110 cm (> 99th centile; *Z*-score + 2.217) and head circumference of 49 cm (25th centile; *Z*-score − 0.683), and minor facial dysmorphism (synopsis, brachycephaly, low anterior and posterior hairline) (Fig. 1A right). External genitalia, hands, feet, and skin were normal. The neurodevelopmental assessment carried out with module 1 of ADOS 2, due to the lack of language skills, gave a high risk for severe autism spectrum disorder (22 points). Brain MRI was normal except for an unspecified delay of myelination. The kidney ultrasound was normal. FMR1 molecular testing, CGH array, and metabolic screening gave negative or normal results.

### Exome findings

Variant filtering assuming de novo and recessive inheritance did not find any candidate genotype in OMIM genes previously associated with related human phenotypes. In detail, the application of the HPO terms ``intellectual disability (HP: 0001249)”, “autism (HP: 0000717)”, and “delayed speech and language development (HP: 0000750)” to the trios data did not identify any pathogenic and likely pathogenic variants in known genes. No pathogenic or likely pathogenic variants following the assumed inheritance patterns were found in the excluded genes, with one or more OMIM disease entries. The list of variants of unknown significance with inconsistent familial transmission is available upon request.

In Individual 1 (MyGene ID: 20047000000), scrutiny of the sequence data of genes not already associated with human diseases identified a heterozygous dinucleotide TC/CT deletion in the exon 15 (c.1570_1571del—equivalent deletions in Supplemental Figure S1B) of the canonical MAP4K4 Ensembl reference transcript model (ENST00000324219.9—NM_001395002.1), predicted to result in a frameshift of the corresponding protein sequence and premature termination (L524Tfs*3). In Individual 2 (DECIPHER ID: 4414689), exome sequencing revealed a heterozygous G nucleotide deletion at one of the GGG nucleotides representing the exon 1 splice donor site (c.57 + 1del—equivalent deletions in Supplemental Figure S1B) of the same transcript model, resulting in the disruption of the donor site itself. Both alternate variants were validated with Sanger sequencing from independent blood drawn (Fig. 1B—additional Sanger validations including families in Supplemental Fig. S1A).

Such alternate variants were not found in parents’ DNA and, therefore, classified as de novo, occurred at conserved residues/nucleotides according to PhastCons100way (score = 1.0), and were absent in GnomAD (Karczewski 2020). The Loss-of-Function Observed/Expected score was 0.093, thus suggesting that MAP4K4 is intolerant to loss of function variants. While in Individual 1, the allele fraction for the alternate allele displays heterozygosity (19 reference counts—25 alternate counts), in Individual 2, the alternate variant showed an allele frequency of just 30.2% (67 reference counts—29 alternate counts). This suggests mosaicism for this MAP4K4 variant in the blood tissue of Individual 2, further confirmed by Sanger analysis (Fig. 1B—additional sanger validations including families in Supplemental Figure S1A)). Both identified variants were considered “pathogenic” according to the ACMG criteria (PVS1_VeryStrong, PS2_Moderate; PM2_Moderate).

### Transcriptomic analysis

**Fig. 2:**
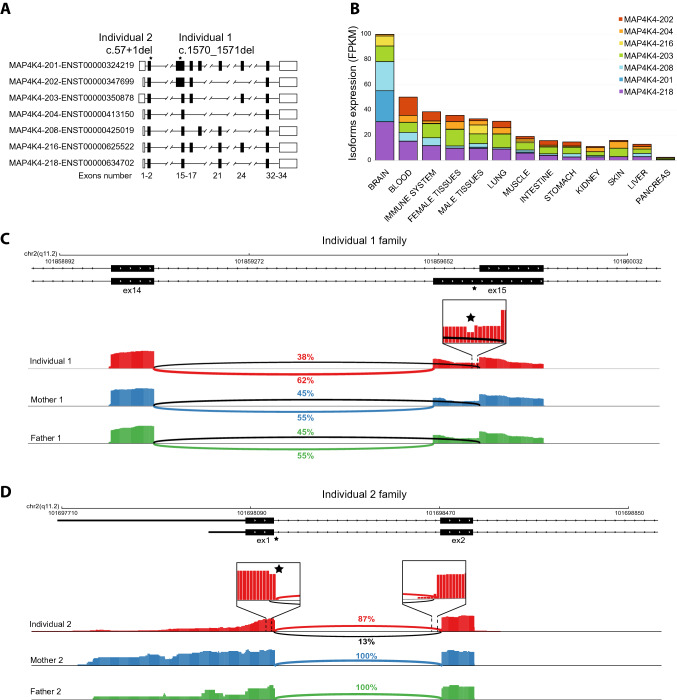
Transcriptomic analyses reveal molecular lesions on MAP4K4 mRNAs. **A** MAP4K4 major isoforms structure from HPA tissues. On top are reported the positions of the identified variants. At the bottom, the number of exons based on MAP4K4 transcript-201. **B** Bar plot showing the expression (FPKM) of the major MAP4K4 isoforms in different tissues. **C**, **D** Genome browser visualization of the MAP4K4 locus at exon 14–15 region in Individual 1 family and exon 1 region in Individual 2 family. Black stars mark the position of the alternate variants. For each member of the family, it is shown the reads coverage from RNA sequencing along with a line connecting the donor and acceptor splice sites. Highlighted for individual 1 are the decreased number of reads in the deletion position. Highlighted for individual 2 is the alternative splicing induced by the deletion. For both individuals, it is shown that the ratio of alternative splicing junctions reported at each splice site. Schematic representation of deleted regions and counts from RNA seq is shown in Supplementary Fig. S2

As MAP4K4 has never been associated with genetic conditions, we sought to investigate its expression pattern, searching for a potential explanation for the observed neurodevelopmental phenotype. MAP4K4 harbors several alternative splicing isoforms which differ for the shuffling of alternative exons in the middle of the gene body (Fig. 2A). According to the HPA database (see methods), MAP4K4 shows a nearly ubiquitous expression level across tissues and cell types, with an enhanced expression in all neuronal derivatives (Fig. 2B). It is possible to appreciate that MAP4K4 alternative splicing isoforms MAP4K4-218, -208, -203 were expressed more ubiquitously, while the MAP4K4-201 isoform displays neuronal specificity. Accordingly, the variant c.57 + 1del is present in the constitutive exon 1, the variant c.1570_1571del is part of an alternative exon 15 isoform that is common to the MAP4K4-201 neuronal isoform, and the MAP4K4-202 non-neuronal isoform. From our analysis, the blood resulted as the second tissue expressing almost all MAP4K4 isoforms, including all exon 1 and exon 15 isoforms. Therefore, we applied transcriptomic analysis to the peripheral blood of both Individuals to support the predicted functional effects of the identified alternate variants.

To this end, we isolated peripheral blood mononuclear cells (PBMC) from blood samples drawn from the affected Individuals, their parents, and siblings and performed full-length mRNA sequencing. Quantitative visualization of the RNA-seq reads allowed us to define how the variants in Individuals affect the splicing pattern and stability of MAP4K4 transcripts (Figs. 2C, D).

In Individual 1, we confirmed by RNA sequencing the de novo dinucleotide deletion in exon 15. This exon is characterized by two distinct splicing isoforms, with the alternate variant present only in the longer isoform (Fig. 2C). As both exon 15 splicing variants (short and long) are common in neuronal and non-neuronal isoforms (Fig. 2B), we proceeded with a qualitative assessment of their relative abundance from RNA extracted from PBMC. From the transcriptomics data, we detected in both parents a balanced expression of the reads (split reads) mapping across the splice junctions of exons 14–15. Such reads start at exon 14 and map at an equal ratio to either the shorter or longer isoform of exon 15 (45% vs 55% respectively, in both parents). Conversely, Individual 1 showed an unbalanced expression of such reads (38% vs 62% respectively). We reasoned that the reduced splicing of the later exon 15 acceptor site could be the result of the identified deletion, which lies in the putative pyrimidine tract, at just 7 bp from the short exon 15 acceptor site. Using MaxEntScan::score3ss (Yeo and Burge 2004), we confirmed that indeed the deletion affects the splicing efficiency of the later exon 15 acceptor site (Supplementary Table 1), favoring the splicing at the earlier exon 15 acceptor site.

We then quantified the expression of reference and alternate alleles at the deletion site. Indeed, through the analysis of genotyped mRNA reads at the deletion site, we distinguished and quantified the reference from the alternate allele, which showed a 93–45 reads ratio, respectively (Supplemental Fig. S2A). Collectively, these data confirmed that the Individual 1 alternate variant is associated with reduced expression of the associated transcript and an alteration of the physiological splicing pattern of exons 14–15.

In Individual 2, we confirmed by RNA sequencing the de novo nucleotide deletion at the splice donor site of exon 1 (Fig. 2D). We observed that while the parents' exon 1 displays a 100% splicing efficiency with the proper splice acceptor in exon 2, Individual 2 displays a novel aberrant alternative splicing. This alternative transcript, containing the alternate allele, was heavily under-expressed compared to the reference spliced isoform at a level that is also much lower than the observed level of mosaicism (13%—Supplemental Fig. S2A shows details of the aberrant splicing and associated RNA counts). Also in this case, using MaxEntScan::score5ss (ref), we confirmed that the deletion affects the splicing efficiency of the exon 1 donor site (Supplementary Table 1), favoring the use of the newly aberrant donor splice site. Consequently, we calculate that this aberrant splicing variant leads to a frameshift transcript and premature termination codon (Asp20Ilefs*51). This aberrant splicing pattern is absent in all the splicing junctions of exons 1–2 from 54 tissues and cells of the GTEx database (Lonsdale 2013).

## Discussion

In this report, we applied diagnostic RNA sequencing in a clinical setting to validate and support original exome data pointing to MAP4K4 as a novel disease-driving gene.

The two individuals under study have been independently admitted for developmental delay and autism spectrum disorder, with or without additional aspecific anomalies (i.e., microcephaly and hypogonadism). While trio analysis of variants in OMIM genes associated with the related HPO terms failed to identify a causative genotype, prioritization of variants in novel genes by inheritance pattern assuming a Mendelian transmission and matching through Matchmaker Exchange (Philippakis et al. 2015) allowed associating the disorder observed in Individual 1 and 2 with de novo frameshift variants in MAP4K4.

Computational analysis revealed that MAP4K4 has a very high neuronal preferential expression. Therefore, truncating variants in this gene could explain neurodevelopmental phenotypes, such as the selective developmental delay and autism spectrum disorder, observed in the two individuals under study.

Nevertheless, MAP4K4 is a ubiquitous gene with appreciable expression levels in various tissues and organs. The significant expression of MAP4K4 in peripheral blood allowed us to apply transcriptomic data on this gene for collecting functional proof of the deleterious effect of the identified alternate variants. More specifically, the analysis of the expression of the MAP4K4 isoforms between brain and peripheral blood shows that the identified alternate variants fell in exons adequately expressed in both tissues. In this scenario, we recognized that the transcriptomic data that we obtained from the peripheral blood of the two individuals were adequate proxies (i.e., “avatars”) for studying a possible deleterious effect of the identified alternate variants in the affected but less accessible organ (i.e., brain). Therefore, it is likely that both alternate variants elicit the same non-physiological alterations of the splicing machinery and NMD in the brain. Accordingly, transcriptomic data in both individuals confirmed predictions showing that all mutated isoforms are less abundant than their reference counterparts as a likely result of NMD.

Previous reports highlighted MAP4K4 as a crucial player in neuronal pathways in vitro (Heyne 2018; Wu et al. 2019) and found MAP4K4 mutated in a cohort of patients with Neurodevelopmental disorders and epilepsy (Heyne 2018). Moreover, a chromosomal deletion of a locus including, among other genes, MAP4K4, was reported in a patient with intellectual disability (Lopes et al. 2019).

Interestingly, Individual 1 also presented hypogonadal hypogonadism and obesity, features not registered in Individual 2. Although we limited this study to two individuals, we could assume that the multi-systemic presentation of Individual 1 is not the chance concurrence of two independent disorders but rather the pleiotropic manifestations of the identified variant. In line with this hypothesis, Danai and collaborators (Danai et al. 2015) showed that inducible deletions of Map4k4 cause obesity and insulin sensitivity in KO mice.

Finally, a de-novo MAP4K4 pathogenic frameshift variant has been found in a fetus with heart and kidney defects (Vora et al. 2017) at prenatal ultrasound scan. Unfortunately, post-therapeutic abortion autopsy findings were not available. Such a case is a clue for pleiotropy extending to the heart and kidneys of null variants in MAP4K4. Besides the endocrinological co-morbidity observed in Individual 1 (see above), we did not find any convincing support for the presumed pleiotropic effect in association with the MAP4K4 null alleles identified here. Given the extremely low number of reported cases with deleterious variants in MAP4K4, the mechanisms underlying such variability remain elusive. Nevertheless, we cannot exclude environmental/non-genetic or extra-locus genetic modifiers, as well as tissue-specific expression of MAP4K4 isoforms as possible explanations for the variable disease expression associated with MAP4K4 null alleles.

Altogether, in this work, we identified MAP4K4 as a candidate for an ultra-rare, Mendelian form of neurodevelopmental disorder. We also demonstrated how transcriptomics could significantly support the clinical interpretation of the molecular findings extracted from exome sequencing. Using an alternate tissue (blood) as a source of genetic material, we demonstrated the feasibility of our strategy in pinpointing disease genes, which are otherwise challenging to identify in the primarily affected tissues.

## Supplementary Information

Below is the link to the electronic supplementary material.

Table 1: MaxEntScan analyses suggest decreased canonical splicing efficiency in tested individuals. Top: MaxEntScan::score3ss reveals lower acceptor splicing sequence efficiency through all the quantifications. MAXENT: Maximum Entropy Model; MM: First-order Markov Model; WMM: Weight Matrix model. Bottom: MaxEntScan::score5ss reveals lower donor splicing sequence efficiency through all the quantifications. MAXENT: Maximum Entropy Model; MM: First-order Markov Model; WMM: Weight Matrix model; MMD: Maximum Dependence Decomposition Model (PDF 391 kb)

Figure S1: A) Sanger sequencing spectra showing heterozygous variant in exon 15 (individual 1) and mosaicism of exon 1 (individual 2) analyzed with Rv and Fw primers and compared with parents spectra (Fw primers). B) Alternative mutations and positions of mutations for Individual 1 (top) and Individual 2 (bottom) deletions. (PDF 832 kb)

Figure S2: A) Schematic representations of the effect of deletions in exon 15 (Individual 1) and in exon 1 (Individual 2). Translation changes are reported under each sequence. For individual 1, the number of reads at deletion positions is reported (PDF 432 kb)

## Data Availability

Exome and transcriptome sequencing data are not publicly available due to patient privacy. Prioritized variants are listed (MyGene ID: 20047000000; DECIPHER ID: 4414689).
