# Supplementary material for: Integrated exome and transcriptome analysis prioritizes MAP4K4 de novo frameshift variants in autism spectrum disorder as a novel disease–gene association
Source: Hum Genet. 2022 Dec 5;142(3):343–50. doi: 10.1007/s00439-022-02497-y (PMC9950172; doi:10.1007/s00439-022-02497-y)
Supplement: Supplementary file 1 — Table 1: MaxEntScan analyses suggest decreased canonical splicing efficiency in tested individuals. Top: MaxEntScan::score3ss reveals lower acceptor splicing sequence efficiency through all the quantifications. MAXENT: Maximum Entropy Model; MM: First-order Markov Model; WMM: Weight Matrix model. Bottom: MaxEntScan::score5ss reveals lower donor splicing sequence efficiency through all the quantifications. MAXENT: Maximum Entropy Model; MM: First-order Markov Model; WMM: Weight Matrix model; MMD: Maximum Dependence Decomposition Model (PDF 391 kb) [file 439_2022_2497_MOESM1_ESM.pdf]

MaxEntScan::score3ss

Sample	MAXENT	MM	WMM
Exon15_WT	9.62	8.96	9.99
Exon15_DelTC	6.52	7.01	7.37

MaxEntScan::score5ss

Sample	MAXENT	MM	WMM	MMD
Exon1_WT	9.89	10.12	9.42	13.98
Exon1_DelG	-14.23	-12.36	-12.57	-8.27
