# Supplementary material for: Integrated exome and transcriptome analysis prioritizes MAP4K4 de novo frameshift variants in autism spectrum disorder as a novel disease–gene association
Source: Hum Genet. 2022 Dec 5;142(3):343–50. doi: 10.1007/s00439-022-02497-y (PMC9950172; doi:10.1007/s00439-022-02497-y)
Supplement: Supplementary file 2 — Figure S1: A) Sanger sequencing spectra showing heterozygous variant in exon 15 (individual 1) and mosaicism of exon 1 (individual 2) analyzed with Rv and Fw primers and compared with parents spectra (Fw primers). B) Alternative mutations and positions of mutations for Individual 1 (top) and Individual 2 (bottom) deletions. (PDF 832 kb) [file 439_2022_2497_MOESM2_ESM.pdf]

A

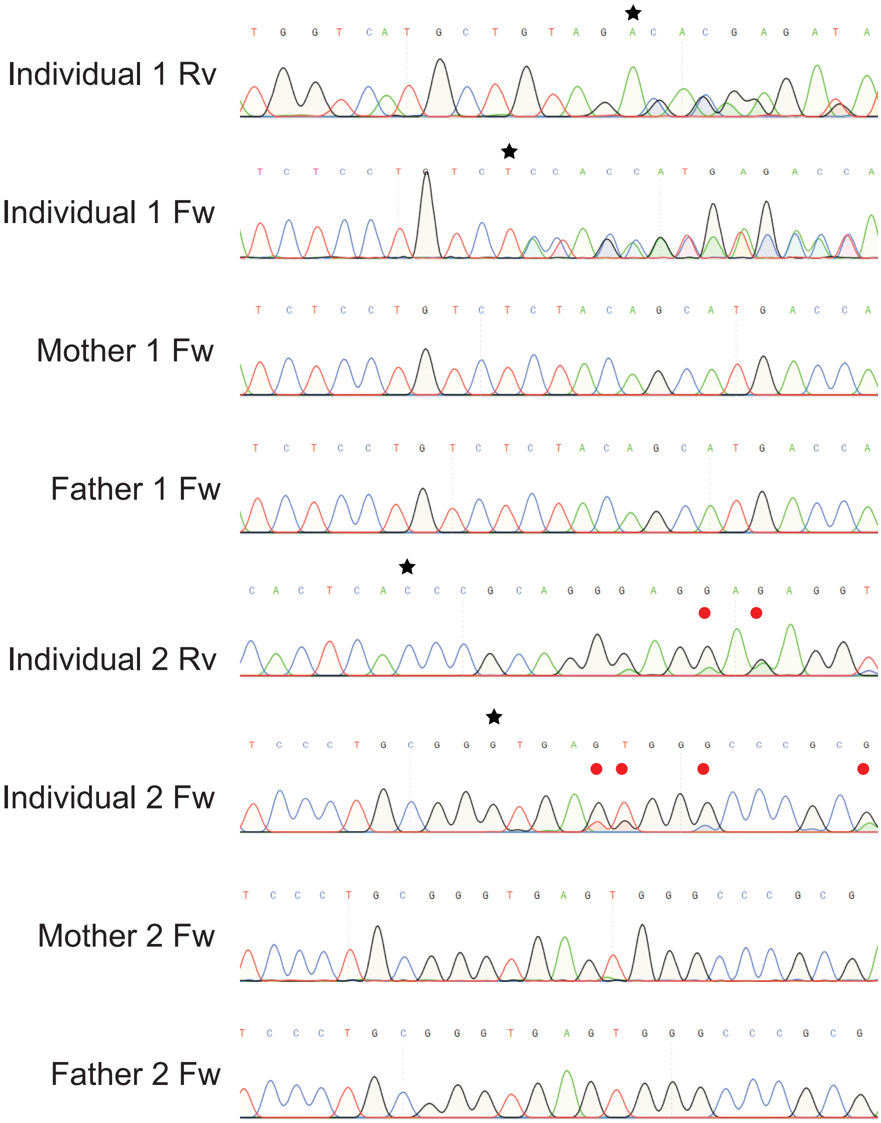

B

Individual 1 alternative mutations

chr-101859727-TC TCCTG~~TC~~TCTACAGC

chr-101859728-CT TCCTGT~~CT~~CTACAGC

chr-101859729-TC TCCTGTC~~TC~~TACAGC

chr-101859730-CT TCCTGTCT~~CT~~ACAGC

Individual 2 alternative mutations

chr-101698136-G CCTGC~~G~~GGTGAGT

chr-101698137-G CCTGCG~~G~~GTGAGT

chr-101698138-G CCTGCGG~~G~~TGAGT
