# Supplementary material for: Integrated exome and transcriptome analysis prioritizes MAP4K4 de novo frameshift variants in autism spectrum disorder as a novel disease–gene association
Source: Hum Genet. 2022 Dec 5;142(3):343–50. doi: 10.1007/s00439-022-02497-y (PMC9950172; doi:10.1007/s00439-022-02497-y)
Supplement: Supplementary file 3 — Figure S2: A) Schematic representations of the effect of deletions in exon 15 (Individual 1) and in exon 1 (Individual 2). Translation changes are reported under each sequence. For individual 1, the number of reads at deletion positions is reported (PDF 432 kb) [file 439_2022_2497_MOESM3_ESM.pdf]

A

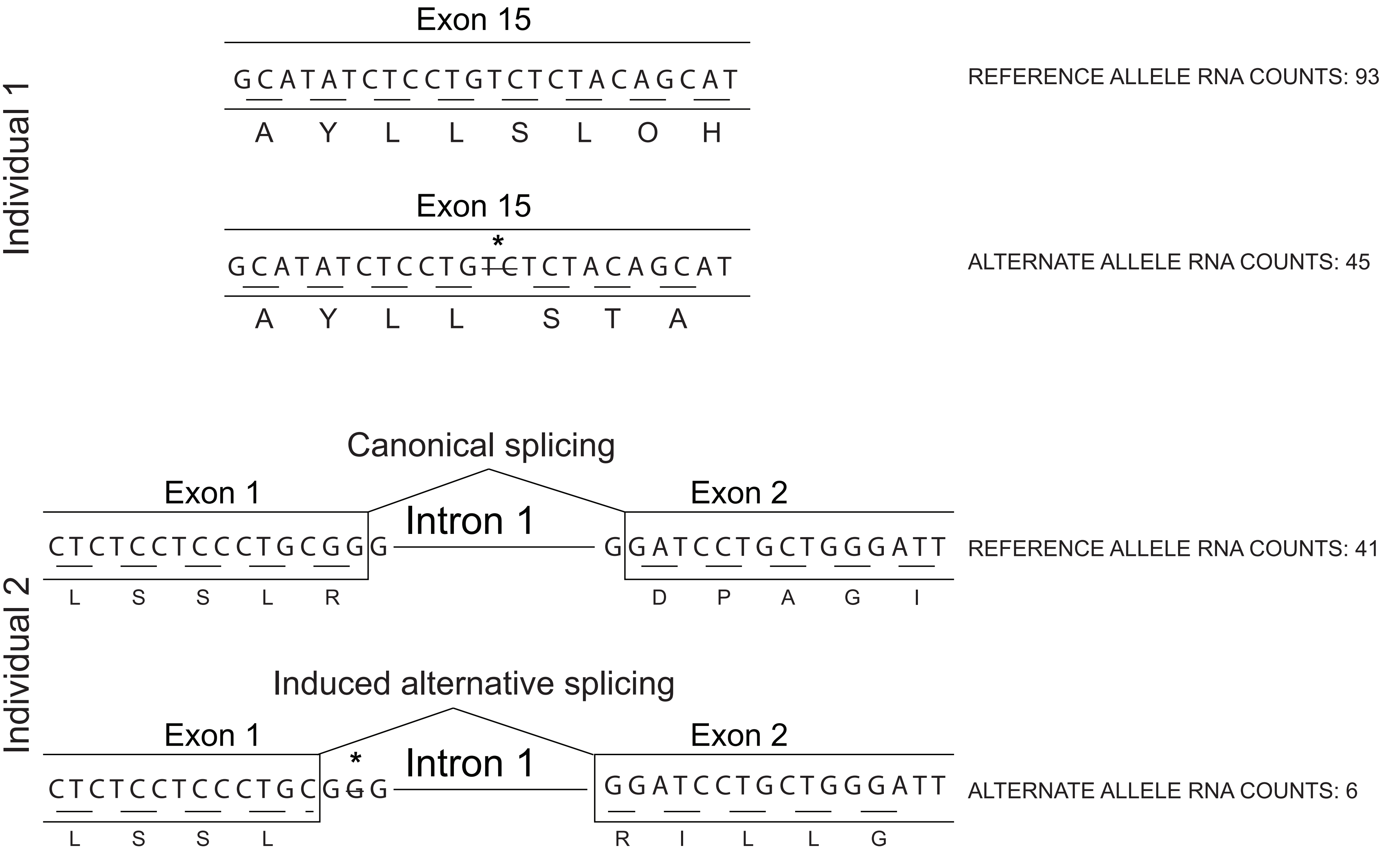

Individual 1
Exon 15
GCATATCTCCTGTCTCTACAGCAT
A Y L L S L O H
REFERENCE ALLELE RNA COUNTS: 93
Exon 15
*
GCATATCTCCTGTCTCTACAGCAT
A Y L L S T A
ALTERNATE ALLELE RNA COUNTS: 45
Individual 2
Canonical splicing
Exon 1
Intron 1
Exon 2
CTCTCCTCCCTGCGGG
GGATCCTGCTGGGATT
L S S L R
D P A G I
REFERENCE ALLELE RNA COUNTS: 41
Induced alternative splicing
Exon 1
*
Intron 1
Exon 2
CTCTCCTCCCTGCGGG
GGATCCTGCTGGGATT
L S S L
R I L L G
ALTERNATE ALLELE RNA COUNTS: 6
